# Neurophysiological brain function predicts response to cognitive rehabilitation and mindfulness in multiple sclerosis: a randomized trial

**DOI:** 10.1007/s00415-024-12183-w

**Published:** 2024-01-26

**Authors:** Ilse M. Nauta, Roy P. C. Kessels, Dirk Bertens, Cornelis J. Stam, Eva E. M. Strijbis, Arjan Hillebrand, Luciano Fasotti, Bernard M. J. Uitdehaag, Hanneke E. Hulst, Anne E. M. Speckens, Menno M. Schoonheim, Brigit A. de Jong

**Affiliations:** 1 MS Center Amsterdam, Neurology, Vrije Universiteit Amsterdam, Amsterdam Neuroscience, Amsterdam UMC Location VUmc, Amsterdam, The Netherlands; 2 Donders Institute for Brain, Cognition and Behaviour, Radboud University, Nijmegen, The Netherlands; 3 Klimmendaal Rehabilitation Center, Arnhem, The Netherlands; 4 Vincent Van Gogh Institute for Psychiatry, Venray, The Netherlands; 5 Department of Medical Psychology, Radboud University Medical Center, Nijmegen, The Netherlands; 6 MEG Center, Clinical Neurophysiology, Vrije Universiteit Amsterdam, Amsterdam Neuroscience, Amsterdam UMC Location VUmc, Amsterdam, The Netherlands; 7 Health, Medical and Neuropsychology Unit, Institute of Psychology, Leiden University, Leiden, The Netherlands; 8 Department of Psychiatry, Radboud University Medical Center, Nijmegen, The Netherlands; 9 MS Center Amsterdam, Anatomy and Neurosciences, Vrije Universiteit Amsterdam, Amsterdam Neuroscience, Amsterdam UMC Location VUmc, Amsterdam, The Netherlands

**Keywords:** Multiple sclerosis, Mindfulness, Cognitive rehabilitation, Functional brain network, Cognitive impairment

## Abstract

**Background:**

Cognitive treatment response varies highly in people with multiple sclerosis (PwMS). Identification of mechanisms is essential for predicting response.

**Objectives:**

This study aimed to investigate whether brain network function predicts response to cognitive rehabilitation therapy (CRT) and mindfulness-based cognitive therapy (MBCT).

**Methods:**

PwMS with cognitive complaints completed CRT, MBCT, or enhanced treatment as usual (ETAU) and performed three measurements (baseline, post-treatment, 6-month follow-up). Baseline magnetoencephalography (MEG) measures were used to predict treatment effects on cognitive complaints, personalized cognitive goals, and information processing speed (IPS) using mixed models (secondary analysis REMIND-MS study).

**Results:**

We included 105 PwMS (96 included in prediction analyses; 32 CRT, 31 MBCT, 33 ETAU), and 56 healthy controls with baseline MEG. MEG did not predict reductions in complaints. Higher connectivity predicted better goal achievement after MBCT (*p* = 0.010) and CRT (*p* = 0.018). Lower gamma power (*p* = 0.006) and higher connectivity (*p* = 0.020) predicted larger IPS benefits after MBCT. These MEG predictors indicated worse brain function compared to healthy controls (*p* < 0.05).

**Conclusions:**

Brain network function predicted better cognitive goal achievement after MBCT and CRT, and IPS improvements after MBCT. PwMS with neuronal slowing and hyperconnectivity were most prone to show treatment response, making network function a promising tool for personalized treatment recommendations.

**Trial registration:**

The REMIND-MS study was prospectively registered in the Dutch Trial registry (NL6285; https://trialsearch.who.int/Trial2.aspx?TrialID=NTR6459).

**Supplementary Information:**

The online version contains supplementary material available at 10.1007/s00415-024-12183-w.

## Introduction

People with multiple sclerosis (PwMS) often experience cognitive problems, both patient-reported complaints and objective impairments, which have profound effects in daily life [1]. PwMS who are most prone to develop objective impairments show functional brain network alterations, including neuronal slowing [2, 3] and shifts in functional connectivity [4] and network integration [5, 6]. There is increasing evidence that behavioral treatments such as cognitive rehabilitation therapy (CRT) [7] and mindfulness-based interventions [8, 9] may ameliorate patient-reported cognitive complaints and objective cognitive function.

However, treatment response for individual PwMS varies greatly, leading to conflicting reports on group effects [10–12]. Preliminary work has shown that PwMS with CRT treatment response seem to have less severe structural [11] and functional [12] network disruptions, especially of the default mode network (DMN). Consequently, it has been postulated that patients whose brain network has not altered below a certain threshold of efficiency may benefit more from cognitive treatments than patients with more severe network disconnection and efficiency loss. This indicates that largest treatment effects are likely achieved during a specific ‘window of opportunity’ [12]. Identification of further neurobiological characteristics that increase the probability of treatment response may help identify patient subgroups that benefit most from cognitive treatments. This may lead to a more careful correspondence between patients’ symptoms and capacities, and the possible interventions to ameliorate cognitive dysfunctions.

We investigated whether functional brain network alterations were related to baseline cognition to confirm their relevance for underlying mechanisms of cognitive impairment. Next, we studied whether these network measures also predicted patients’ responses to CRT and mindfulness-based cognitive therapy (MBCT). Previous results from this randomized controlled trial (RCT; REMIND-MS study) [13] indicated positive effects of both CRT and MBCT on patient-reported complaints, CRT on personalized cognitive goals, and of MBCT on information processing speed (IPS, i.e., the most commonly affected cognitive domain in MS [1]). We therefore specifically investigated response on these outcome measures in relation to functional brain network properties. Network function was quantified using magnetoencephalography (MEG), as it offers a more direct measure of neuronal activity than, for instance, functional MRI [14], and MEG-based network measures have previously been linked to cognitive decline in MS [6].

## Materials and methods

### Study design, participants, and procedure

**Fig. 1:**
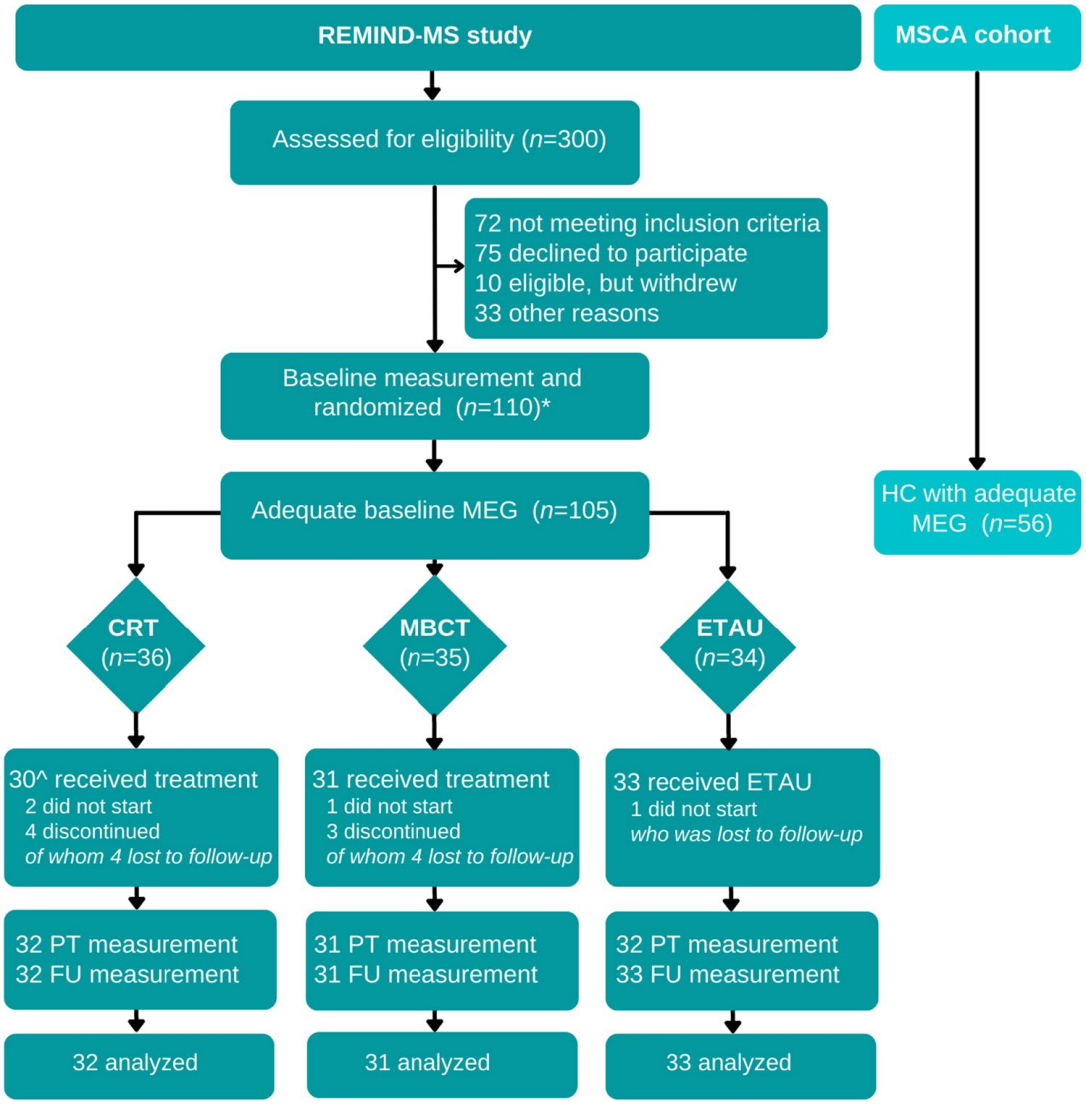
Flowchart of the study. *The complete flowchart, detailed reasons of exclusion and missing data of the REMIND-MS study have been reported in Nauta et al. [13] ^Two patients who did not complete the treatment did return for follow-up visits, leading to 32 measurements in this group. *REMIND-MS* cognitive rehabilitation and mindfulness in MS, *MSCA* Multiple Sclerosis Center Amsterdam, *MEG* magnetoencephalography, *CRT* cognitive rehabilitation therapy, *MBCT* mindfulness-based cognitive therapy, *ETAU* enhanced treatment as usual, *HC* healthy control, *PT* post-treatment, *FU* 6-month follow-up

The protocol of the REMIND-MS study [15] and effects on cognitive outcomes have been published previously [13]. In summary, the REMIND-MS study is an RCT that compared CRT and MBCT to enhanced treatment as usual (ETAU). Patients were included at the MS Center Amsterdam and Klimmendaal Rehabilitation Center in Arnhem. Following baseline measurements, patients were randomized for each center separately into CRT, MBCT, or ETAU, using a block size of 6 and 9 and a minimization procedure (equal weights for factors cognitive complaints, age and sex). Treatments took place at both centers and assessments were performed at the MS Center Amsterdam at three time points (i.e., baseline, post-treatment, 6-month follow-up). Assessors were blinded to treatment allocation. Participants were included if they had a verified MS diagnosis, were aged 18–65 at inclusion, and reported cognitive complaints (scoring ≥ 23 on the Multiple Sclerosis Neuropsychological Questionnaire—Patient version) [16]. Participants were excluded if they had previous experience with the interventions offered, a severe psychiatric disorder (psychosis or suicidal ideation), an inability to speak or read Dutch, or physical or cognitive disabilities, comorbidities, or treatments that would interfere too much with the interventions to enroll in the study. From the included patients in the REMIND-MS study [13], only patients with adequate baseline MEG data were included in this article. Additionally, MEG data of healthy controls (HCs) of the Amsterdam MS Cohort were retrospectively used to analyze baseline MEG differences. Figure 1 presents the flowchart. All participants gave written informed consent prior to participation.

### Treatments

Details of the treatments have been published previously [15]. In short, both CRT and MBCT lasted nine weeks. CRT consisted of 9 weekly 2.5-h sessions in closed groups of 3–6 patients. During CRT, patients were taught compensatory strategies for IPS, memory, executive function, and mental fatigue. CRT additionally focused on emotional and behavioral changes and grief resolution. MBCT consisted of 8 weekly 2.5-h sessions in groups of 4–7 patients and a 6-h silent day. Patients were trained to self-regulate attention and to be aware of present-moment emotions, thoughts, and automatic behavioral patterns, and patients were encouraged to develop more adaptive ways of responding to their experiences, including their illness. Patients performed homework assignments for CRT and guided mindfulness exercises for MBCT (30–45 min, 6 days/week). ETAU consisted of treatment as usual, and additionally of one individual appointment with an MS specialist nurse who focused on psycho-education. Similar psycho-educative information was also provided during CRT and MBCT.

### Cognitive outcomes

The primary outcome of the REMIND-MS trial [13, 15] was the Cognitive Failures Questionnaire (CFQ) [17], which measures patient-reported cognitive complaints. The Behavior Rating Inventory of Executive Function–Adult Version (BRIEF-A) measured patient-reported complaints in executive functioning [18]. Two personalized cognitive goals were determined by the patients at baseline using Goal Attainment Scaling (GAS), which concerned problems in daily life due to their cognitive difficulties. The outcome levels for each goal corresponded to a 6-point Likert scale (e.g., expected level, more than expected, less than expected). Scores were transformed into standardized t-scores and averaged across goals [19]. Four objective cognitive domains were investigated with an adapted Minimal Assessment of Cognitive Function in MS (MACFIMS) [20]: (1) IPS (Symbol Digit Modalities Test, Stroop Color-Word Test I and II), (2) memory (California Verbal Learning Test, the Brief Visuospatial Memory Test-Revised), (3) visuospatial processing (Judgment of Line Orientation Test), (4) executive function (verbal fluency, Stroop Color–Word Test interference score, Delis–Kaplan Executive Function System Sorting Test). At baseline, raw scores were corrected for the effects of age, sex, and education and transformed into z-scores, both based on a normative sample of Dutch HCs (see [21] for a more detailed description of the applied methods). Performance was accordingly classified as cognitively impaired (CI  ≥ 20% of the corrected *z* scores ≥ 1.5 SDs below means of this normative sample of HCs) or cognitively preserved (CP; the remainder). To analyze intervention effects, raw test scores at each time point were converted into *z* scores based on whole-group means and SDs of the REMIND-MS sample and averaged into domain-specific *z* scores [13].

### MEG recording and pre-processing

**Fig. 2:**
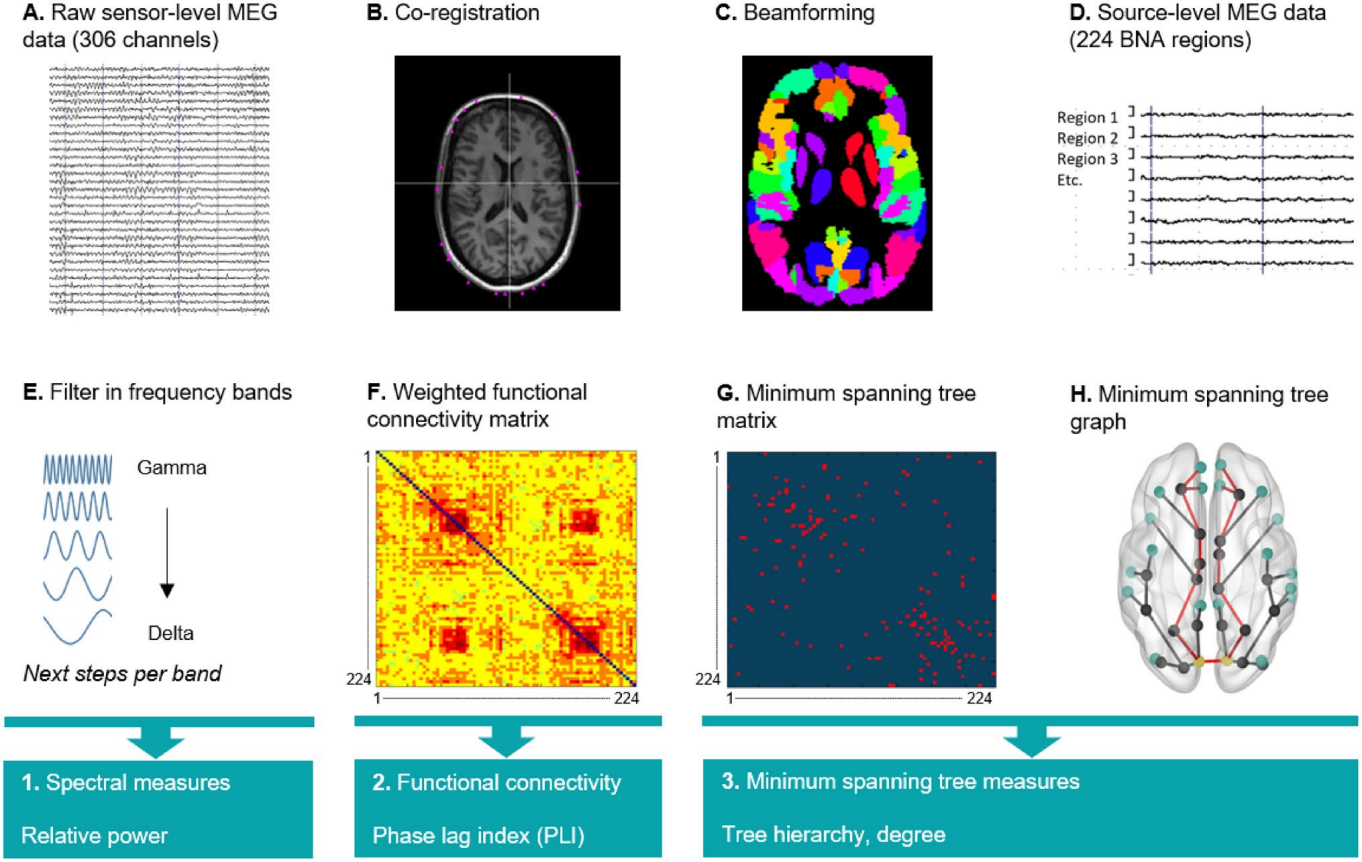
MEG pre-processing steps. **A** Raw MEG recording at sensor level, where each trace represents one MEG channel. Vertical lines mark 1 s segments of data. **B** The MEG recording was co-registered to a surface-matched structural MRI template. **C** Beamforming was applied to convert the MEG signal to source space: signals were projected onto the Brainnetome (BNA) atlas. **D** Each MEG signal corresponds to the centroid of one BNA region (i.e., source level). **E** Data were filtered into six frequency band, and each of the following steps was calculated for each frequency band. **F** The phase lag index (PLI) was calculated between each of 224 regions of the BNA atlas. **G** The Minimum Spanning Tree (MST) was constructed based on the PLI, which consists of the 223 strongest connections. These connections were subsequently binarized. **H** An example of an MST graph. *MEG* magnetoencephalography, *BNA* Brainnetome

Eyes-closed resting-state MEG recordings were performed with a 306-channel whole-head MEG system (Elekta Neuromag Oy, Helsinki, Finland). Acquisition and processing followed a standardized procedure (described previously [6], Fig. 2). Malfunctioning channels were discarded (maximum of 12) after visual inspection and artifacts were removed with the temporal extension of Signal Space Separation [22]. MEG data were co-registered to matched magnetic resonance imaging templates of the scalp surface, and subsequently source localized to the centroids of 224 regions (i.e., 210 cortical and 14 subcortical; Supplemental eTable 1) of the Brainnetome atlas [23] using a beamforming approach [24]. For each participant, 60 consecutive epochs (i.e., based on the participant with lowest number of epochs available) of 3.27 s were filtered into the following frequency bands in Matlab (version R2012a, Mathworks, Natick, MA, USA): delta (0.5–4 Hz), theta (4–8 Hz), alpha1 (8–10 Hz), alpha2 (10–13 Hz), beta (13–30 Hz), and gamma (30–48 Hz).

### MEG measures

Each measure was computed for each frequency band in Matlab using in-house codes [25] and the Brain Connectivity Toolbox. Relative power represents the amount of neuronal activity within each frequency band relative to broad band (0.5–48 Hz). Functional connectivity, the communication strength between regions, was estimated with the phase lag index (PLI) between each pair of regions, both globally and between the DMN (Yeo atlas-based [26], Supplementary Information) and the rest of the brain [27]. Network organization was investigated for the minimum spanning tree (MST), which represents the dichotomized functional backbone of the network [28]. The MST includes a fixed number of regions and connections, and consequently, there are no arbitrary thresholds, which optimizes comparability between participants [28]. The MST was computed with 224 regions (consistent to number of included BNA regions) and the 223 strongest functional connections (based on PLI). MST tree hierarchy (TH) was calculated, representing network efficiency (network integration versus hub overload) [28]. DMN centrality represented the number of MST connections (i.e., degree) within the DMN.

### Sample size calculation

As reported previously [13, 15], treatment effects on the CFQ indicated 33 patients per group (*α* = 0.05, power = 0.80, intra-class correlation = 0.06) and 40 patients per group including drop-out and loss to follow-up.

### Statistical analyses

Statistical analyses were performed in SPSS-26 and STATA-14. First, general linear model analyses were performed to compare baseline MEG measures between HCs, CI, and CP patients (*α* = 0.05). Significant group effects were pairwise compared (three analyses; adjusted *α* = 0.017). In MS, Pearson’s partial correlations were calculated between MEG measures and both patient-reported cognitive complaints (CFQ and two BRIEF-A indices) and objective cognitive function (four domains) (six frequency bands; adjusted *α* = 0.008). Significant (*α* = 0.05) MEG measures were selected as potential baseline moderators of treatment response. Linear mixed-model analyses were subsequently performed with time (post-treatment, 6-month follow-up) as within-subjects factor, treatment (CRT vs. ETAU, MBCT vs. ETAU) as between-subjects factor, and the CFQ, GAS, and IPS as outcome measures. Additionally, a MEG moderator-by-group-by-time interaction was inserted and the moderator-by-group interaction indicated moderation effects (Bonferroni-corrected α based on number of frequency bands included). All analyses were corrected for age, education, and sex, and prediction analyses also for baseline CFQ or IPS (depending on outcome; GAS baseline values are identical by default).

## Results

Of 110 inclusions, five had a low-quality or failed MEG scan at baseline. The remaining 105 patients (75% women, age 48.6 ± 10.0, 56% CI) were included in our baseline analyses, together with 56 HCs (61% women, age 47.8 ± 9.7) from the Amsterdam MS cohort. Of 105 patients, 96 (32 CRT, 31 MBCT, 33 ETAU) had at least one follow-up measurement and were included in the predictive analyses. Figure 1 represents the flowchart and Table 1 shows the participants’ characteristics.

**Table 1:** Demographics and disease-related characteristics per group at baseline

	Healthy controls and cognitive groups at baseline			Treatment groups		
	HCs (*n* = 56)	CP-MS (*n* = 46)	CI-MS (*n* = 59)	CRT (*n* = 32)	MBCT (*n* = 31)	ETAU (*n* = 33)
Demographics						
Age (years), mean (SD)	47.8 (9.7)	47.2 (9.6)	49.7 (10.2)	50.9 (7.8)	46.2 (10.3)	49.2 (10.5)
Sex (women), *n* (%)	34 (61%)	39 (85%)	40 (68%)	24 (75%)	22 (71%)	26 (79%)
Education (high),^a^ *n* (%)	31 (55%)	25 (54%)	37 (63%)	20 (63%)	20 (65%)	19 (58%)
Disease-related characteristics						
MS type (RRMS/SPMS/PPMS/unclear^b^), %	Not applicable	78/9/9/4	58/22/15/5	63/16/16/6	58/23/13/7	70/15/12/3
Disease duration (years),^c^ median (IQR)	Not applicable	10.9 (16.1)	17.1 (18.1)	15.6 (12.7)	9.3 (16.6)	14.2 (22.5)
EDSS, median (range)	Not applicable	3.5 (2.0–6.0)	4.0 (2.0–7.5)	3.75 (2.0–6.5)	3.5 (2.0–7.0)	4.0 (2.5–7.5)

*HCs* healthy controls, *CP-MS* cognitively preserved multiple sclerosis patients, *CI-MS* cognitively impaired multiple sclerosis patients, *CRT* cognitive rehabilitation therapy, *MBCT* mindfulness-based cognitive therapy, *ETAU* enhanced treatment as usual, *RRMS* relapsing–remitting multiple sclerosis, *SPMS* secondary progressive multiple sclerosis; *PPMS* primary progressive multiple sclerosis, *EDSS* Expanded Disability Status Scale

^a^Education was coded according to the Dutch educational Verhage classification and categorized as low (i.e., completed average-level secondary education or lower; levels 1–5) or high (i.e., completed high-level secondary education or university degree; levels 6–7). ^b^’Unclear’ indicates that the MS type could not be specified by the neurologist. ^c^Disease duration represents the time between the first onset of neurological complaints and the visit date

### Summary treatment effect

As previously published [13], patient-reported complaints were reduced after CRT and MBCT compared to ETAU after treatment completion, but not 6 months later. At 6-month follow-up, CRT had a positive effect on personalized cognitive goals and MBCT on IPS compared to ETAU.

### Baseline group differences in MEG

**Fig. 3:**
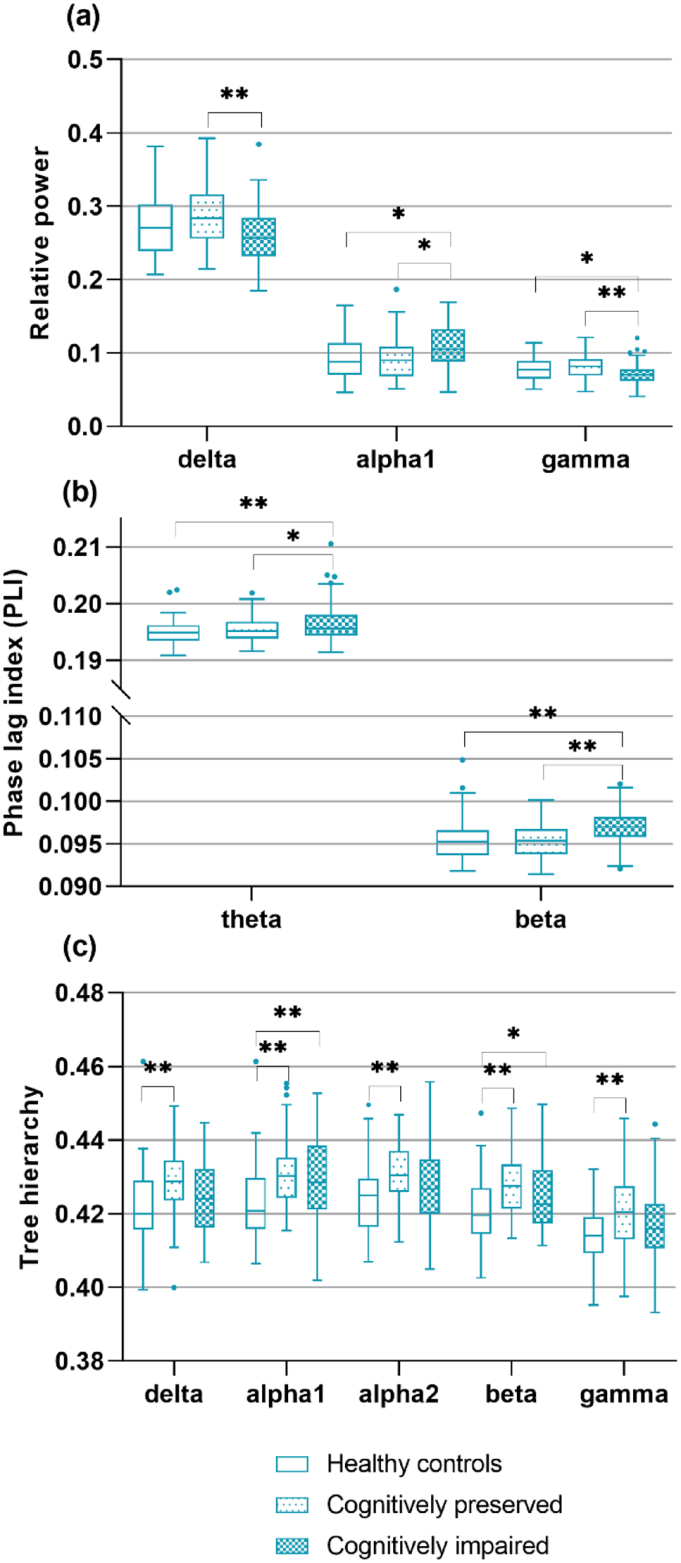
Group comparisons between cognitive groups and healthy controls. The significant main group effects and subsequent pairwise comparisons are depicted in this Figure. **a** Relative power per group. **b** Functional connectivity (phase lag index) per group. **c** Tree hierarchy per group. *p < 0.05, **p < 0.0167 (multiple comparison correction)

Delta (*p* = 0.015), alpha1 (*p* = 0.029), and gamma (*p* = 0.005) relative power differed between groups (Table 2; Fig. 3): CI patients had lower delta (*p* = 0.004) and gamma power (*p* = 0.002) than CP patients. CI and CP patients did not differ from HCs. Theta (*p* = 0.006) and beta (*p* = 0.002) PLI differed between groups (Table 2; Fig. 3): CI patients had higher theta (*p* = 0.002) and beta (*p* = 0.006) PLI than HCs and higher beta PLI than CP patients (*p* = 0.001). Regionally, similar group effects for theta (*p* = 0.018) and beta (*p* = 0.001) PLI-DMN were found: CI patients had higher theta (*p* = 0.007) and beta (*p* = 0.003) DMN-PLI than HCs (*p* = 0.003), and higher beta DMN-PLI than CP patients (*p* = 0.001). Delta (*p* = 0.023), alpha1 (*p* = 0.001), alpha2 (*p* = 0.049), beta (*p* = 0.008), and gamma (*p* = 0.015) network integration (TH) differed between groups (Table 2; Fig. 3): CI patients had higher alpha1 TH than HCs (*p* = 0.002). CP patients had higher delta (*p* = 0.006), alpha1 (*p* = 0.002), alpha2 (*p* = 0.014), beta (*p* = 0.003), and gamma (*p* = 0.004) TH than HCs. DMN centrality showed no group differences.

**Table 2:** Comparison of MEG measures between cognitive groups and healthy controls

	HC	CP	CI	Group difference
	*M* (SD)	*M* (SD)	*M* (SD)	*P* value
Spectral measures				
Power delta	0.272 (0.039)	0.285 (0.042)	0.259 (0.043)	0.015*^c^
Power theta	0.155 (0.032)	0.156 (0.031)	0.165 (0.045)	0.223
Power alpha1	0.095 (0.030)	0.094 (0.030)	0.108 (0.028)	0.029*
Power alpha2	0.106 (0.022)	0.101 (0.027)	0.108 (0.023)	0.447
Power beta	0.293 (0.043)	0.282 (0.047)	0.289 (0.058)	0.414
Power gamma	0.078 (0.015)	0.082 (0.018)	0.071 (0.015)	0.005*^c^
Functional connectivity				
PLI delta	0.229 (0.004)	0.230 (0.004)	0.231 (0.005)	0.109
PLI theta	0.195 (0.002)	0.195 (0.002)	0.197 (0.004)	0.006*^b^
PLI alpha1	0.281 (0.004)	0.280 (0.004)	0.281 (0.004)	0.801
PLI alpha2	0.230 (0.005)	0.229 (0.005)	0.229 (0.005)	0.475
PLI beta	0.096 (0.003)	0.095 (0.002)	0.097 (0.002)	0.002*^b,c^
PLI gamma	0.096 (0.002)	0.096 (0.002)	0.096 (0.002)	0.744
Network integration				
TH delta	0.422 (0.010)	0.428 (0.010)	0.424 (0.009)	0.023*^a^
TH theta	0.423 (0.009)	0.428 (0.009)	0.425 (0.008)	0.057
TH alpha1	0.423 (0.011)	0.431 (0.010)	0.429 (0.011)	0.001*^a,b^
TH alpha2	0.424 (0.009)	0.430 (0.009)	0.427 (0.010)	0.049*^a^
TH beta	0.421 (0.010)	0.428 (0.008)	0.425 (0.010)	0.008*^a^
TH gamma	0.414 (0.009)	0.421 (0.011)	0.417 (0.009)	0.015*^a^

*Significant main group effect, which are represented in bold (*p* < 0.05). The following groups differed using a Bonferroni-corrected α (*p* < 0.0167): ^a^HC and CP, ^b^HC and CI, ^c^CP and CI

### Correlations between cognition and MEG at baseline in MS

Within MS, baseline patient-reported cognitive complaints were unrelated to any of the MEG measures (*p* > 0.008). Regarding objective cognitive function, worse memory performance was related to lower delta (*r* = 0.26, *p* = 0.007), higher alpha1 (*r* = − 0.34, *p* < 0.001), and lower gamma (*r* = 0.26, *p* = 0.008) power. Worse memory (*r* = − 0.27, *p* = 0.006) and executive function (*r* = − 0.27, *p* = 0.006) were also related to higher theta DMN-PLI (DMN strength). Furthermore, worse memory and IPS were associated with lower beta (*r* = 0.32, *p* = 0.001) and gamma TH (*r* = 0.30, *p* = 0.002), respectively.

### MEG predictors of treatment response to CRT or MBCT in MS

**Fig. 4:**
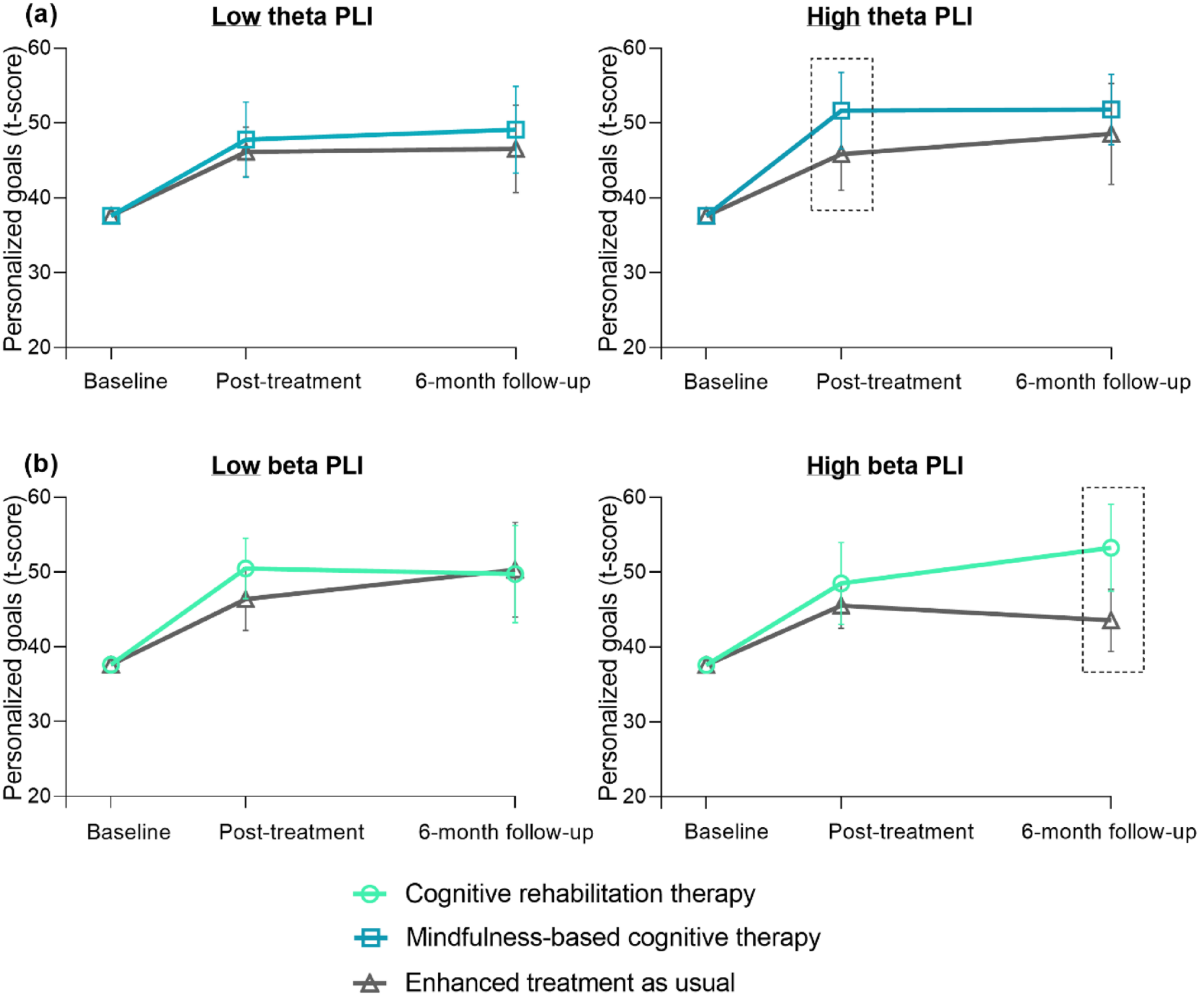
Visualization of MEG treatment predictors on personalized goals. Treatment response is visualized in patients with low and high baseline values (using a median split) of significant MEG predictors. The dotted rectangles highlight the treatment effects predicted by MEG measures. **a** Response on personalized goals visualized in patients with low theta PLI (left) and high theta PLI (right) at baseline. **b** Response on personalized goals visualized in patients with low beta PLI (left) and high beta PLI (right) at baseline, the same pattern was found in the default mode network. *PLI* phase lag index; *DMN* default mode network

**Fig. 5:**
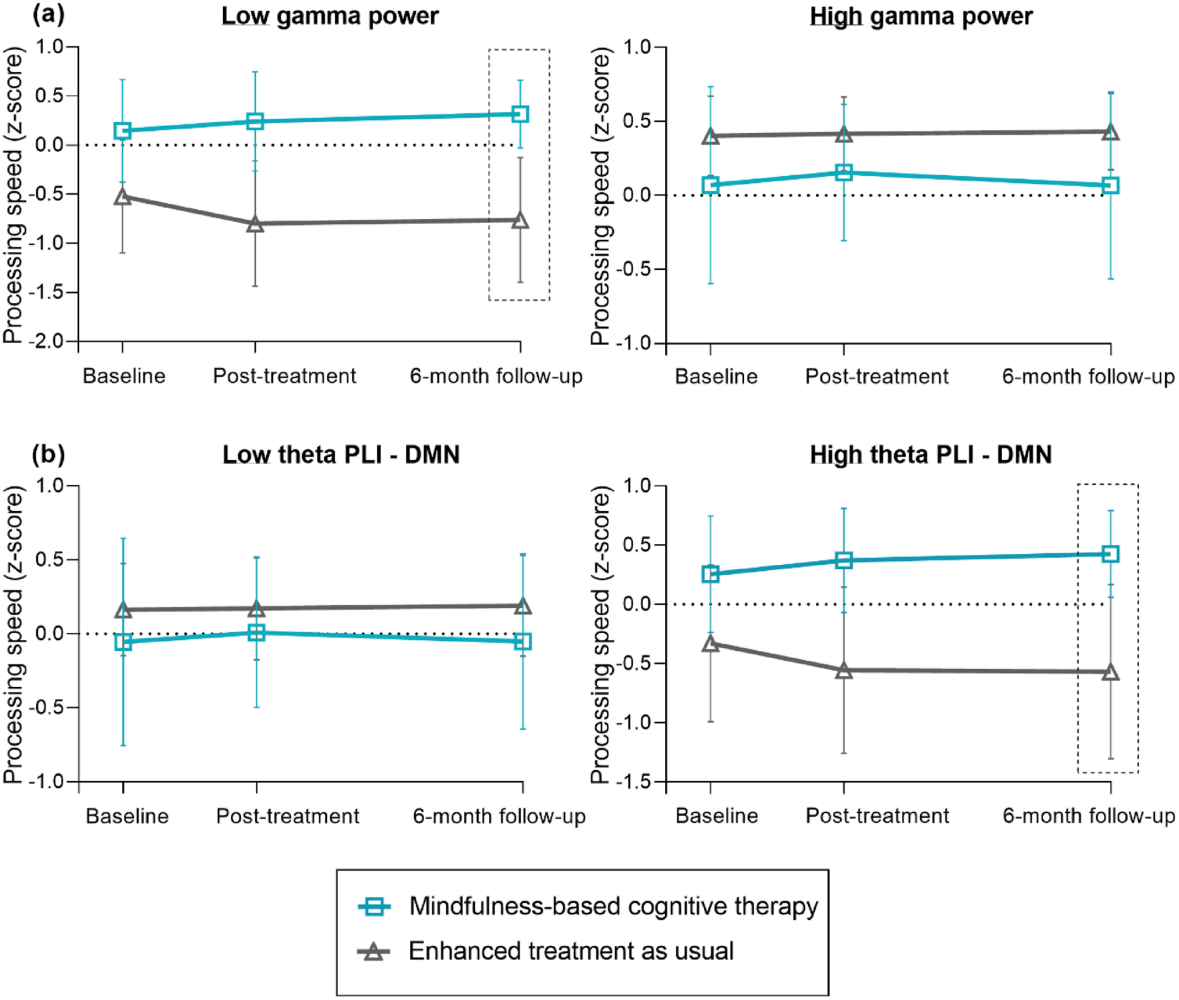
Visualization of MEG treatment predictors on information processing speed. Treatment response is visualized in patients with low and high baseline values (using a median split) of significant MEG predictors. The dotted rectangles highlight the treatment effects predicted by MEG measures. **a** Response on processing speed visualized in patients with low gamma power (left) and high gamma power (right) at baseline. **b** Response on processing speed visualized in patients with low theta PLI in the DMN (left) and high theta PLI in the DMN (right) at baseline. *PLI* phase lag index, *DMN* default mode network

Tables 3, 4, and 5 show the potential baseline MEG measures and results of the prediction analyses. No predictors were found for reductions in patient-reported cognitive complaints (*p* > 0.05; Table 3). Regarding personalized goals (Table 4; Fig. 4), patients with higher beta PLI, both global (*p* = 0.018) and in the DMN (*p* = 0.022), showed greater benefits 6 months after CRT. Patients with higher theta PLI benefited more on personalized goals immediately following MBCT (*p* = 0.010). Regarding IPS, patients with lower gamma power (*p* = 0.006) and higher theta DMN-PLI (*p* = 0.020) benefited more on IPS 6 months after MBCT (Table 5; Fig. 5). No predictors were found for CRT.

**Table 3:** MEG treatment moderators on patient-reported cognitive complaints

	CRT vs. ETAU				MBCT vs. ETAU			
	Post-treatment		6-month follow-up		Post-treatment		6-month follow-up	
	*β* (95% CI)	*p*	*β* (95% CI)	*p*	*β* (95% CI)	*p*	*β* (95% CI)	*p*
Spectral								
Power delta	67.8 (− 28.1, 163.7)	0.166	40.8 (− 55.1, 136.7)	0.405	85.1 (− 50.8, 221.0)	0.220	13.1 (− 123.6, 149.8)	0.851
Power theta	64.6 (− 45.3, 174.5)	0.249	42.7 (− 66.9, 152.4)	0.445	− 8.8 (− 146.7, 129.2)	0.901	1.3 (− 136.6, 139.1)	0.986
Power alpha1	− 64.6 (− 231.4, 102.2)	0.448	6.3 (− 159.9, 172.6)	0.940	− 63.9 (− 267.8, 139.9)	0.539	− 27.0 (− 230.9, 177.0)	0.796
Power alpha2	− 43.0 (− 229.5, 143.6)	0.652	9.3 (− 177.3, 195.8)	0.923	− 41.8 (− 248.9, 165.3)	0.692	60.6 (− 153.1, 274.3)	0.579
Power beta	− 83.6 (− 172.8, 5.6)	0.066	− 76.7 (− 165.8, 12.5)	0.092	− 49.6 (− 148.8, 49.7)	0.328	− 49.6 (− 149.1, 49.9)	0.329
Power gamma	147.0 (− 137.0, 431.0)	0.310	8.1 (− 275.7, 291.9)	0.955	− 22.1 (− 328.9, 284.8)	0.888	− 9.5 (− 316.0, 297.1)	0.952
Connectivity								
PLI theta	561.1 (− 830.3, 1952.5)	0.429	− 217.4 (− 1599.1, 1164.4)	0.758	64.0 (− 2254.4, 2382.3)	0.957	− 12.4 (− 2323.2, 2298.4)	0.992
PLI beta	− 1253.8 (3182.1, 674.4)	0.202	− 770.8 (− 2699.0, 1157.4)	0.433	− 1067.1 (− 3467.6, 1333.5)	0.384	− 1129.4 (− 3529.7, 1270.8)	0.356
PLI DMN theta	602.6 (− 831.1, 2036.2)	0.410	− 15.1 (− 1441.7, 1411.4)	0.983	− 294.8 (− 2604.5, 2015.0)	0.802	− 402.5 (− 2706.3, 1901.3)	0.732
PLI DMN beta	− 1453.4 (− 3411.1, 504.3)	0.146	− 1071.5 (− 3028.9, 885.8)	0.283	− 910.8 (− 3497.1, 1675.4)	0.490	− 815.0 (− 3400.4, 1770.3)	0.537
Integration								
TH delta	219.3 (− 277.8, 716.5)	0.387	389.4 (− 102.7, 881.5)	0.121	228.4 (− 297.7, 754.5)	0.395	− 100.8 (− 622.6, 421.1)	0.705
TH alpha1	− 253.0 (− 764.5, 258.5)	0.332	− 43.6 (− 553.3, 466.0)	0.867	28.5 (− 460.2, 517.3)	0.909	− 271.2 (− 760.5, 218.1)	0.277
TH alpha2	15.9 (− 504.7, 536.6)	0.952	140.0 (− 369.3, 649.3)	0.590	109.4 (− 418.5, 637.3)	0.685	− 87.6 (− 604.2, 429.0)	0.740
TH beta	− 359.2 (− 895.2, 176.7)	0.189	− 37.9 (− 570.1, 494.4)	0.889	436.3 (− 213.1, 1085.7)	0.188	420.8 (− 226.7, 1068.2)	0.203
TH gamma	-56.9 (− 530.3, 416.4)	0.814	107.9 (− 360.5, 576.3)	0.652	− 386.0 (− 968.3, 196.3)	0.194	− 334.7 (− 916.4, 247.1)	0.260

*CRT* cognitive rehabilitation therapy, *MBCT* mindfulness-based cognitive therapy, *ETAU* enhanced treatment as usual, *PLI* phase lag index, *DMN* default mode network, *TH* tree hierarchy

Treatment moderators of the effects on patient-reported cognitive complaints (i.e., Cognitive Failures Questionnaire (CFQ)), corrected for age, education, sex, and baseline CFQ scores. *Significant moderator *p* < 0.05. **Significant moderator after multiple comparison correction: power *p* < 0.008 (corrected for six frequency bands), PLI *p* < 0.025 (corrected for two frequency bands), TH *p* < 0.01 (corrected for five frequency bands)

**Table 4:** MEG treatment moderators on personalized goals

	CRT vs. ETAU				MBCT vs. ETAU			
	Post-treatment		6-month follow-up		Post-treatment		6-month follow-up	
	*β* (95% CI)	*p*	*β* (95% CI)	*p*	*β* (95% CI)	*p*		*p*
Spectral								
Power delta	3.0 (− 93.0, 99.1)	0.950	− 90.4 (− 186.4, 5.6)	0.065	− 82.8 (− 200.1, 34.5)	0.167	− 82.5 (− 199.8, 34.8)	0.168
Power theta	− 55.0 (− 163.6, 53.5)	0.320	33.9 (− 74.3, 142.1)	0.539	43.0 (− 78.8, 164.9)	0.489	**126.7 (5.2, 248.2)**	0.041*
Power alpha1	− 51.2 (− 205.2, 102.9)	0.515	125.1 (− 28.4, 278.5)	0.110	**172.4 (10.4, 334.3)**	0.037*	**197.4 (35.9, 358.8)**	0.017*
Power alpha2	34.5 (− 156.0, 225.0)	0.723	− 117.9 (− 308.4, 72.6)	0.225	49.7 (− 140.3, 239.6)	0.608	− 144.8 (− 334.8, 45.2)	0.135
Power beta	26.2 (− 64.4, 116.7)	0.571	39.7 (− 50.8, 130.2)	0.390	− 8.6 (− 97.3, 80.1)	0.849	− 17.2 (− 105.8, 71.4)	0.703
Power gamma	197.3 (− 78.3, 472.8)	0.161	− 183.6 (− 459.0, 91.8)	0.191	− 23.5 (− 293.7, 246.7)	0.865	− 68.4 (− 338.4, 201.6)	0.620
Connectivity								
PLI theta	− 342.2 (− 1612.8, 928.4)	0.598	446.1 (− 816.2, 1708.5)	0.489	**2491.9 (589.0, 4394.9)**	0.010**	**2095.1 (198.6, 3991.6)**	0.030*
PLI beta	131.5 (− 1705.1, 1968.0)	0.888	**2224.6 (388.3, 4061.0)**	0.018**	1019.5 (− 1048.8, 3087.8)	0.334	1549.8 (− 518.1, 3617.7)	0.142
PLI DMN theta	− 504.0 (− 1816.2, 808.2)	0.452	431.8 (− 874.3, 1737.9)	0.517	**2193.1 (255.2, 4131.0)**	0.027*	1748.3 (− 184.7, 3681.2)	0.076
PLI DMN beta	− 33.3 (− 1903.8, 1837.1)	0.972	**2181.5 (311.7, 4051.2)**	0.022**	1023.8 (− 1205.8, 3253.4)	0.368	1380.8 (− 848.0, 3609.6)	0.225
Integration								
TH delta	171.9 (− 329.1, 672.9)	0.501	− 89.3 (− 583.7, 405.1)	0.723	− 178.9 (− 648.2, 290.3)	0.455	− 88.2 (− 553.1, 376.7)	0.710
TH alpha1	262.9 (− 234.3, 760.1)	0.300	− 89.2 (− 584.1, 405.6)	0.724	− 261.1 (− 684.7, 162.6)	0.227	− 380.7 (− 803.1, 41.7)	0.077
TH alpha2	236.4 (− 277.9, 750.8)	0.368	− 132.2 (− 632.1, 367.6)	0.604	− 329.9 (791.0, 131.3)	0.161	− 396.3 (− 844.7, 52.0)	0.083
TH beta	152.6 (− 362.8, 667.9)	0.562	**514.4 (3.5, 1025.3)**	0.048*	− 488.2 (− 1058.5, 82.0)	0.093	− 491.0 (− 1059.8, 77.8)	0.091
TH gamma	373.7 (− 82.4, 829.8)	0.108	38.4 (− 411.5, 488.4)	0.867	− 4.9 (− 524.5, 514.6)	0.985	− 175.9 (− 692.0, 340.1)	0.504

*CRT* cognitive rehabilitation therapy, *MBCT* mindfulness-based cognitive therapy, *ETAU* enhanced treatment as usual, *PLI* phase lag index, *DMN* default mode network, *TH* tree hierarchy

Treatment moderators of the effects on personalized cognitive goals (GAS), corrected for age, education, and sex. Bold represents significant moderators (either before or after correction).*Significant moderator *p* < 0.05. **Significant moderator after multiple comparison correction: power *p* < 0.008 (corrected for six frequency bands), PLI *p* < 0.025 (corrected for two frequency bands), TH *p* < 0.01 (corrected for five frequency bands)

**Table 5:** MEG treatment moderators on information processing speed

	CRT vs. ETAU				MBCT vs. ETAU			
	Post-treatment		6-month follow-up		Post-treatment		6-month follow-up	
	*β* (95% CI)	*p*	*β* (95% CI)	*p*	*β* (95% CI)	*p*	*β* (95% CI)	*p*
Spectral								
Power delta	0.8 (− 2.8, 4.4)	0.655	1.3 (− 2.4, 4.9)	0.492	− 0.9 (− 6.1, 4.3)	0.736	− 1.5 (− 6.8, 3.8)	0.579
Power theta	− 0.6 (− 4.6, 3.3)	0.752	2.8 (− 1.2, 6.9)	0.173	4.5 (− 0.9, 9.9)	0.102	**5.6 (0.2, 11.1)**	0.044*
Power alpha1	− 0.9 (− 6.9, 5.2)	0.782	1.3 (− 4.9, 7.5)	0.691	4.5 (− 3.0, 12.0)	0.242	**8.6 (0.9, 16.2)**	0.028*
Power alpha2	0.9 (− 6.1, 7.9)	0.798	− 4.5 (− 11.5, 2.5)	0.211	4.9 (− 3.6, 13.5)	0.260	1.5 (− 7.2, 10.1)	0.736
Power beta	− 0.7 (− 4.0, 2.6)	0.686	− 2.3 (− 5.7, 1.0)	0.175	− 2.8 (− 6.7, 1.2)	0.167	− 2.9 (− 6.9, 1.0)	0.148
Power gamma	− 0.5 (− 10.9, 9.9)	0.929	− 2.5 (− 13.0, 8.1)	0.645	− 11.2 (− 22.8, 0.4)	0.059	− **16.5 (**− **28.3, **− **4.7)**	0.006**
Connectivity								
PLI theta	− 18.6 (− 65.8, 28.5)	0.439	8.7 (− 39.1, 56.5)	0.720	30.2 (− 56.4, 116.8)	0.494	**89.7 (1.6, 177.8)**	0.046*
PLI beta	− 11.8 (− 80.9, 57.3)	0.737	− 10.6 (− 79.9, 58.6)	0.763	24.4 (− 70.9, 119.6)	0.616	54.8 (− 40.8, 150.5)	0.261
PLI DMN theta	− 10.4 (− 59.4, 38.7)	0.678	14.1 (− 35.8, 63.9)	0.580	44.1 (− 42.6, 130.9)	0.319	**104.7 (16.2, 193.2)**	0.020**
PLI DMN beta	− 1.7 (− 71.9, 68.5)	0.962	− 9.5 (− 79.9, 60.9)	0.791	36.5 (− 64.9, 137.9)	0.481	63.3 (− 38.7, 165.3)	0.224
Integration								
TH delta	− 8.4 (− 26.4, 9.5)	0.359	− 13.3 (− 31.5, 4.9)	0.152	− 10.0 (− 30.0, 10.1)	0.329	− **24.8 (**− **45.3, **− **4.4)**	0.017*
TH alpha1	1.7 (− 17.1, 20.5)	0.858	− 10.8 (− 29.7, 8.1)	0.264	− 4.0 (− 23.1, 15.1)	0.683	− 14.1 (− 33.5, 5.2)	0.153
TH alpha2	− 5.1 (− 24.3, 14.1)	0.600	− 9.1 (− 28.1, 10.0)	0.352	− 11.6 (32.0, 8.8)	0.264	− 19.5 (− 40.0, 0.9)	0.061
TH beta	-1.2 (-20.8, 18.4)	0.903	-6.5 (-26.2, 13.1)	0.515	-0.5 (-25.9, 24.8)	0.967	-3.5 (-29.4, 22.4)	0.791
TH gamma	8.4 (-8.6, 25.5)	0.333	1.6 (-15.4, 18.6)	0.856	-7.4 (-30.2, 15.4)	0.527	-18.7 (-41.6, 4.2)	0.110

Treatment moderators of the effects on information processing speed, corrected for age, education, sex, and baseline information processing speed. Bold represents significant moderators (either before or after correction). *Significant moderator *p* < 0.05. **Significant moderator after multiple comparison correction: power *p* < 0.008 (corrected for six frequency bands), PLI *p* < 0.025 (corrected for two frequency bands), TH *p* < 0.01 (corrected for five frequency bands). *CRT*  cognitive rehabilitation therapy, *MBCT* mindfulness-based cognitive therapy, *ETAU* enhanced treatment as usual, *PLI* phase lag index, *DMN* default mode network, TH = tree hierarchy

### Post hoc cognitive and disability predictors of treatment response

MEG measures predicting treatment response (e.g., higher theta and beta connectivity, lower gamma power) were also identified as abnormal in CI patients at baseline. We therefore also investigated whether baseline objective cognition (i.e., four domains and CI-CP categorization) and disability (Expanded Disability Status Scale [EDSS]) predicted treatment response. Consistent with our previous publication (with a slightly different sample) [13], patients with better baseline IPS showed larger reductions on the CFQ at post-treatment (*β* [95% CI] = − 6.0 [− 10.9, − 1.1]*, p* = 0.016), but neither baseline objective cognition nor disability predicted response after CRT. Regarding personalized goal achievements, baseline cognition and disability did not predict response (*p* > 0.05). For IPS improvement, both cognition and disability predicted response after MBCT, but not after CRT. More specifically, patients with worse IPS (*β* [95% CI] = − 0.3 [− 0.5, − 0.1]*, p* = 0.002), visuospatial function (*β* [95% CI] = − 0.2 [− 0.4, -0.03]*, p* = 0.022), and executive function (*β* [95% CI] = − 0.4 [− 0.7, − 0.1]*, p* = 0.003) at baseline improved more on IPS directly following MBCT. IPS (*β* [95% CI] = − 0.3 [− 0.5, − 0.1]*, p* = 0.001) and visuospatial function (*β* [95% CI] = − 0.2 [− 0.4, − 0.03]*, p* = 0.025) had similar predictive effects at 6-month follow-up, and higher EDSS at baseline also predicted larger effects on IPS at 6-month follow-up (*β* [95% CI] = 0.2 [0.1, 0.3]*, p* = 0.005). Given the predictive value of cognition and disability, the analysis on the predictive value of MEG measures for IPS response after MBCT (i.e., only those significant after correction: gamma power and theta PLI in the DMN) was repeated while also including cognition and EDSS as covariates, showing that MEG measures remained significant response predictors (*p* < 0.05)  of treatments response (see Supplementary Information).

### Post hoc explorations of the tree hierarchy

All significant results that concerned TH were further explored as it represents a balance between the leaf fraction (network integration) and maximum betweenness centrality (hub overload). The findings with the tree hierarchy (*p* < 0.05) were therefore repeated for the leaf fraction and maximum betweenness centrality separately. Group differences and correlations with objective cognitive function were only found for the leaf fraction: CI and CP patients had a higher leaf fraction compared to HCs (delta, theta, alpha1, beta, and gamma; *p* < 0.05). Within the MS patients, lower leaf fraction was related to worse objective cognition (delta, alpha1, beta and gamma; *p* < 0.05). Regarding the prediction analyses, lower delta leaf fraction was a predictor of IPS response after MBCT at 6-month follow-up (*p* = 0.021), whereas higher beta maximum betweenness centrality predicted the post-treatment response on the GAS after CRT (*p* = 0.041).

## Discussion

This study investigated whether MEG-derived functional network measures predicted cognitive treatment response. The presence of neuronal slowing and higher functional connectivity best predicted better achievements of personalized cognitive goals after both CRT and MBCT and larger benefits on IPS after MBCT. Cognitive impairments in MS were related to neuronal slowing, higher connectivity, and a less integrated brain network, while compared to healthy controls, overall PwMS showed more integration and hub-like network topology.

Neuronal slowing is commonly seen in cognitive impaired patients [2, 3], and in our study this was especially represented by *more* alpha1 activity and *less* gamma activity. Contrasting this pattern of neuronal slowing, less delta activity was related to worse cognitive function, which was reported previously in MS [29], also in relation to more structural brain damage [3]. Furthermore, a consistent increase in functional connectivity across frequency bands was seen in CI patients. This may represent a state of hyperexcitability due to damaged inhibitory neurons and/or a loss of inhibitory synapses [30]. Previous studies report higher *and* lower connectivity in relation to cognitive impairment, which may depend on disease stages (e.g., extent of white matter damage) and methodology (e.g., connectivity measure) [4, 31]. Regarding network organization, cognitively preserved PwMS had a *better* integrated and hub-like network organization compared to HCs, whereas within MS, *less* network integration was related to worse cognition. Potentially, patients’ functional brain networks first become more integrated (e.g., functional connections may be redirected toward hub regions, caused by disconnections in existing paths due to structural pathology), eventually leading to a larger burden on hub regions. As MS progresses, these hubs get overloaded, causing the network to ‘collapse’ and to become less integrated [32]. Similar U-shaped patterns in functional network alterations have been reported in patients at risk of Alzheimer’s disease [33], but should be further explored in longitudinal MS studies. Combined, network alterations in the form of neuronal slowing, increased functional connectivity, and increased network integration characterize the brain networks of CI-PwMS.

After confirming their relevance for cognition, these resting-state brain measures were subsequently used to predict treatment response. MEG measures could not predict reductions in patient-reported cognitive complaints, but predicted the response on personalized cognitive goals and IPS. Patients with higher functional connectivity levels prior to treatment onset showed larger responses regarding personalized cognitive goal achievements (beta and theta, after CRT and MBCT) and IPS benefits (theta, after MBCT). Patients with lower gamma power also showed larger IPS benefits after MBCT. These findings indicate that neurobiological markers associated with impaired baseline cognition (i.e., higher connectivity levels, neuronal slowing) also predict larger cognitive treatment effects. This suggests that more affected PwMS are most likely to achieve their personalized goals after both treatments and to improve their objective cognitive function after MBCT. In line with these findings, cognitively and physically more affected patients showed greater benefits on IPS after MBCT, but not on cognitive goal achievements. This could suggest that brain network alterations induced cognitive and physical disabilities, which in turn predicted treatment effects. Interestingly, further explorations showed that brain network characteristics predicted treatment response at least partially independent of cognition and disability.

Our findings that more severely affected patients (regarding network alteration, cognitive impairments and disability levels) showed the largest improvements are in contrast with previous studies in which *less* affected patients were found to benefit most from CRT. However, the present study is the first to also include neurophysiological measures of brain function [10–12]. Importantly, this field remains new as only few studies have investigated predictors of cognitive treatment response in MS, which differ from our study in terms of methodology and sample characteristics, including disease duration [11] and percentage of CI patients [12]. Despite these methodological differences, the discrepancy indicates that different cognitive treatment types are not uniformly effective in similar MS subsamples. Potentially, MBCT is more effective in severely affected PwMS than CRT, as it is an experiential training where patients implicitly learn new techniques through practice [34]. Once PwMS are cognitively more affected, engaging in an intervention that heavily relies on cognitive strategies (i.e., compensatory CRT) or cognitive effort (i.e., restorative CRT, such as solving complex cognitive puzzles) may be challenging for some patients, making an experientially based approach particularly suitable to improve their objective cognitive function. Interestingly, the concept of cognitively or physically more advanced patients responding better was not a universal finding in our data, as we previously reported [13] that *better* cognitive function predicted larger reductions in patient-reported cognitive complaints after MBCT. This suggests that the treatments can be beneficial across a wide severity spectrum, yet it seems to depend on the level of symptom severity which outcome measures are most likely to improve. Although the exact reasons behind these contradictions remain unanswered, this finding highlights that patient-reported cognitive complaints and objective cognitive impairments are two different constructs with potentially different underlying mechanisms: patient-reported complaints in general correlate weakly with objective impairments, but are more often associated with other psychological problems, such as depressed mood or anxiety [35]. In line with this, we found that MEG-derived measures of brain function were related to objective cognitive function, but not to patient-reported cognitive complaints.

This study has some methodological considerations. Functional brain networks can be characterized with a variety of measures. To limit the number of analyses, specific network measures were selected based on their previously shown relation with cognition in MS. Potentially, important network measures were missed, as cross-sectional correlates of cognitive impairment are not necessarily the best predictors of future changes in cognition [6], although this remains unclear for treatment effects. Similarly regarding resting-state networks, we only examined the DMN, as it is important for cognition [36]. This limits our conclusions: based on our findings, we did not see a larger or smaller effect in the DMN compared to the global network. The DMN possibly plays an important role when investigating connectivity changes following MBCT, as meditation and mind-wandering, both important concepts within the mindfulness training, have been associated with DMN connectivity [37], but this needs further exploration. Furthermore, we used MEG given its high temporal resolution and found that MEG measures predicted treatment effects in MS, which highlights their potential clinical relevance. However, MEG scans are not routinely performed in clinical practice due to its limited availability and high cost. It would be valuable to replicate these findings in clinical electroencephalography (EEG) as well as functional MRI. Lastly, our findings indicate which groups of patients are most likely to improve after the treatments. It is important to mention that patients who do not match these characteristics may still benefit from the treatments to a certain extent. Future studies should also investigate treatment predictors on an individual level.

To conclude, PwMS who were more severely affected, in the form of more severe functional network disruptions, worse cognitive function, and high disability levels, benefited most from mindfulness with regard to IPS. Patients with increased functional connectivity levels, reflecting a more severely affected brain network, achieved their personalized goals better after both cognitive rehabilitation and mindfulness. In contrast, patients with better cognition showed larger reductions in patient-reported cognitive complaints after mindfulness, further stressing the differentiation between objective and patient-reported cognition. As such, network function, together with baseline cognition and patients’ individual treatment goals (e.g., which functions they aim to improve), could play an important role in predicting treatment response and personalized treatment recommendations.

### Supplementary Information

Below is the link to the electronic supplementary material.

Supplementary file1 (PDF 71 KB)

## Data Availability

Anonymized data, not published in the article, will be shared upon reasonable request from a qualified investigator.
