# Supplementary material for: Neurophysiological brain function predicts response to cognitive rehabilitation and mindfulness in multiple sclerosis: a randomized trial
Source: J Neurol. 2024 Jan 26;271(4):1649–62. doi: 10.1007/s00415-024-12183-w (PMC10972975; doi:10.1007/s00415-024-12183-w)
Supplement: Supplementary file 1 — Supplementary file1 (PDF 71 KB) [file 415_2024_12183_MOESM1_ESM.pdf]

Supplemental eTable 1. Brainnetome regions[1] included in this study

Lobe	Gyrus	ID Left hemisphere	ID Right hemisphere	Abbreviation and description of area
Frontal Lobe	Superior Frontal Gyrus	1	2	A8m, medial area 8
		**3**	**4**	A8dl, dorsolateral area 8
		**5**	**6**	A9l, lateral area 9
		7	8	A6dl, dorsolateral area 6
		9	10	A6m, medial area 6
		**11**	**12**	A9m, medial area 9
		**13**	**14**	A10m, medial area 10
	Middle Frontal Gyrus	15	16	A9/46d, dorsal area 9/46
		17	18	IFJ, inferior frontal junction
		19	20	A46, area 46
		21	22	A9/46v, ventral area 9/46
		**23**	24	A8vl, ventrolateral area 8
		25	26	A6vl, ventrolateral area 6
		**27**	28	A10l, lateral area 10
	Inferior Frontal Gyrus	29	30	A44d,dorsal area 44
		31	32	IFS, inferior frontal sulcus
		**33**	34	A45c, caudal area 45
		**35**	36	A45r, rostral area 45
		37	38	A44op, opercular area 44
		**39**	40	A44v, ventral area 44
	Orbital Gyrus	**41**	**42**	A14m, medial area 14
		**43**	**44**	A12/47o, orbital area 12/47
		45	46	A11l, lateral area 11
		47	48	A11m, medial area 11
		49	50	A13, area 13
		**51**	**52**	A12/47l, lateral area 12/47
	Precentral Gyrus	53	54	A4hf, area 4 (head and face region)
		55	56	A6cdl, caudal dorsolateral area 6
		57	58	A4ul, area 4 (upper limb region)
		59	60	A4t, area 4 (trunk region)
		61	62	A4tl, area 4 (tongue and larynx region)
		63	64	A6cvl, caudal ventrolateral area 6
	Paracentral Lobule	65	66	A1/2/3ll, area1/2/3 (lower limb region)
		67	68	A4ll, area 4, (lower limb region)

Temporal Lobe	Superior Temporal Gyrus	69	70	A38m, medial area 38
		71	72	A41/42, area 41/42
		73	74	TE1.0 and TE1.2
		75	76	A22c, caudal area 22
		77	78	A38l, lateral area 38
		**79**	80	A22r, rostral area 22
	Middle Temporal Gyrus	**81**	**82**	A21c, caudal area 21
		**83**	**84**	A21r, rostral area 21
		85	86	A37dl, dorsolateral area37
		**87**	**88**	aSTS, anterior superior temporal sulcus
	Inferior Temporal Gyrus	89	90	A20iv, intermediate ventral area 20
		91	92	A37elv, extreme lateroventral area37
		93	94	A20r, rostral area 20
		**95**	96	A20il, intermediate lateral area 20
		97	98	A37vl, ventrolateral area 37
		99	100	A20cl, caudolateral of area 20
		101	102	A20cv, caudoventral of area 20
	Fusiform Gyrus	103	104	A20rv, rostroventral area 20
		105	106	A37mv, medioventral area 37
		107	108	A37lv, lateroventral area 37
	Parahippocampal Gyrus	109	110	A35/36r, rostral area 35/36
		111	112	A35/36c, caudal area 35/36
		**113**	114	TL, area TL (lateral PPHC, posterior parahippocampal gyrus)
		115	116	A28/34, area 28/34 (EC, entorhinal cortex)
		117	118	TI, area TI (temporal agranular insular cortex)
		119	120	TH, area TH (medial PPHC)
	posterior Superior Temporal Sulcus	**121**	**122**	rpSTS, rostroposterior superior temporal sulcus
		**123**	124	cpSTS, caudoposterior superior temporal sulcus
Parietal Lobe	Superior Parietal Lobule	125	126	A7r, rostral area 7
		127	128	A7c, caudal area 7
		129	130	A5l, lateral area 5
		131	132	A7pc, postcentral area 7
		133	134	A7ip, intraparietal area 7 (hIP3)
	Inferior Parietal Lobule	135	136	A39c, caudal area 39 (PGp)
		137	138	A39rd, rostrodorsal area 39 (Hip3)
		139	140	A40rd, rostrodorsal area 40 (PFt)

		**141**	142	A40c, caudal area 40 (PFm)
		**143**	**144**	A39rv, rostroventral area 39 (PGa)
		145	146	A40rv, rostroventral area 40 (PFop)
	Precuneus	147	148	A7m, medial area 7 (PEp)
		149	150	A5m, medial area 5 (PEm)
		**151**	152	dmPOS, dorsomedial parieto-occipital sulcus (PEr)
		**153**	**154**	A31, area 31 (Lc1)
	Postcentral Gyrus	155	156	A1/2/3ulhf, area 1/2/3 (upper limb, head and face region)
		157	158	A1/2/3tonIa, area 1/2/3 (tongue and larynx region)
		159	160	A2, area 2
		161	162	A1/2/3tru, area1/2/3 (trunk region)
Insular Lobe	Insular Gyrus	163	164	G, hypergranular insula
		**165**	166	vIa, ventral agranular insula
		167	168	dIa, dorsal agranular insula
		169	170	vId/vIg, ventral dysgranular and granular insula
		171	172	dIg, dorsal granular insula
		173	174	dId, dorsal dysgranular insula
Limbic Lobe	Cingulate Gyrus	**175**	**176**	A23d, dorsal area 23
		177	**178**	A24rv, rostroventral area 24
		**179**	180	A32p, pregenual area 32
		**181**	**182**	A23v, ventral area 23
		183	184	A24cd, caudodorsal area 24
		185	186	A23c, caudal area 23
		**187**	**188**	A32sg, subgenual area 32
Occipital Lobe	MedioVentral Occipital Cortex	189	190	cLinG, caudal lingual gyrus
		191	192	rCunG, rostral cuneus gyrus
		193	194	cCunG, caudal cuneus gyrus
		195	196	rLinG, rostral lingual gyrus
		197	198	vmPOS,ventromedial parietooccipital sulcus
	lateral Occipital Cortex	199	200	mOccG, middle occipital gyrus
		201	202	V5/MT+, area V5/MT+
		203	204	OPC, occipital polar cortex
		205	206	iOccG, inferior occipital gyrus
		207	208	msOccG, medial superior occipital gyrus
		209	210	lsOccG, lateral superior occipital gyrus
	Amygdala	211	212	mAmyg, medial amygdala

Subcortical areas	Hippocampus	215	216	rHipp, rostral hippocampus
	Basal Ganglia	221	222	GP, globus pallidus
		223	224	NAC, nucleus accumbens
		227	228	dCa, dorsal caudate
		229	230	dlPu, dorsolateral putamen
	Thalamus	245	246	lPFtha, lateral pre-frontal thalamus

Note. This table represents the regions from the Brainnetome (BNA) atlas that were analyzed in this study. The regions included in the default mode network (DMN) are presented in bold, based on Yeo et al.[2]

REFERENCES

1. Fan L, Li H, Zhuo J, et al. The Human Brainnetome Atlas: A New Brain Atlas Based on Connectional Architecture. *Cereb Cortex* 2016; 26: 3508-3526. 20160526. DOI: 10.1093/cercor/bhw157.
2. Yeo TW, De Jager PL, Gregory SG, et al. A second major histocompatibility complex susceptibility locus for multiple sclerosis. *Ann Neurol* 2007; 61: 228-236. 2007/01/26. DOI: 10.1002/ana.21063.
